# Pelvic pedicled omental flaps and autologous free omental grafts in a rabbit model

**DOI:** 10.1186/2054-7099-1-3

**Published:** 2015-04-21

**Authors:** Amelia P Bailey, Amy K Schutt, Lisa M Pastore, Dale W Stovall

**Affiliations:** 5 Department of Obstetrics and Gynecology, 75 Francis Street, Boston, MA 02115 USA; 6 Division of Reproductive Endocrinology and Infertility, University of Virginia, 1215 Lee Street, Charlottesville, VA 22903 USA

**Keywords:** Adhesion prevention, Adhesion barrier, Free graft, Omentum, Pedicled flap

## Abstract

**Background:**

There is a need to identify an inexpensive, effective method to prevent postoperative adhesion formation. The objective of this study was to create a novel model for studying omentum as a pelvic adhesion barrier.

Randomized, prospective, controlled surgical intervention with serial follow-up in 16 female rabbits at a University-based Center for Comparative Medicine. Interventions included bilateral hysterotomy incision and repair. The left hysterotomy was randomized into coverage with an omental flap or graft; the right hysterotomy remained uncovered. Adhesions were scored via laparoscopy on postoperative days 2, 4, 8, and 12; postmortem evaluation and scoring took place on postoperative day 16. Statistical tests consisted of Kappa tests of agreement between adhesion scorers and Kruskal-Wallis nonparametric tests for the comparison of adhesion scores by intervention arm and by uterine horn.

**Results:**

All omental flaps and grafts survived. The only significant difference in mean adhesion scores was seen in non-hysterotomy-associated adhesions, where grafts had a lower score than flaps (p = 0.03).

**Conclusions:**

Survival of all omental flaps and grafts demonstrates that this is a practical model for studying omentum as a pelvic adhesion barrier. Determining the efficacy of omentum as a pelvic adhesion barrier will require further investigation.

## Background

Every experienced surgeon has encountered adhesions in the operating room. Adhesions vary histologically and may be filmy, dense, vascular or avascular. The presence of adhesions intraoperatively often mandates extensive adhesiolysis for completion of many obstetric and gynecologic procedures. Furthermore, adhesions are a significant cause of morbidity including infertility, chronic pain, and bowel obstruction. Significant data have been published with regard to the use of various methods of adhesion prevention, including peritoneal infusions and synthetic adhesion barriers. These data have demonstrated variable success in reducing adhesion formation, and many of these methodologies are associated with significant financial costs. Therefore, there is still a need to identify an inexpensive, effective method to prevent adhesion formation.

Omentum is an intra-abdominal fold of visceral peritoneum that is easily accessible during pelvic surgery and has the potential to be an excellent barrier to prevent adhesions [1]. Omental tissue has already been successfully used in thoracic [2], oncologic [3–7], vascular [2, 8], general [9, 10], and reconstructive surgery [7]. Its unique characteristics include high concentrations of thromboplastin for hemostasis [2, 11] and a trophic effect on surrounding tissues, allowing a free graft to survive in the peritoneal cavity. The omentum also possesses extensive vascularity and is capable of rapid angiogenesis and capillary ingrowth [12, 13]. Moreover, affixing omentum to denuded surfaces of canine bowel and bladder has been shown to prevent scarring and adhesion formation [14].

Free omental grafts have been useful also as vascular patches in animals [8]. When utilized for this purpose, they do not retain their native vascular supply after transposition but have been observed to establish vascular communications with pericardium and various peritoneal structures by quickly invading pleural and serosal surfaces [11]. Graft size is important, though; studies have shown marked necrosis of larger grafts and minimal necrosis of thin grafts in canines after four days [15]. Pedicled omental flaps do maintain their blood supply, thus decreasing the potential for ischemic necrosis. In humans, flaps have even been used to cover and strengthen intestinal and colonic anastomoses [14, 16], in breast reconstruction [9, 10], and to reduce complications after pelvic lymphadenectomy [3]. Yet the use of omentum in gynecologic, specifically uterine, surgery has not been well-investigated. Therefore, the primary purpose of this pilot study was to develop an animal model that can be used to study the utility of the omentum in pelvic surgery. Secondarily, we wanted to investigate the effectiveness of two different omentum-derived adhesion barriers, an autologous free omental graft and a pedicled omental flap, in the prevention of postoperative adhesions.

## Methods

This was a randomized, prospective, controlled surgical intervention with serial follow-up conducted at a University-based Center for Comparative Medicine. Based on an established animal model [17], we obtained sixteen sexually mature New Zealand white rabbits weighing 4-5 kg from a commercial source (Burleson Enterprises, Inc., Unionville, Virginia). The rabbits were observed for six days to allow for acclimation and assessment of health. The animals were maintained on a Teklad Global 2031 high-fiber rabbit diet (Harlan, Madison, Wisconsin) and water ad libitum. Approval for the study was obtained from the Animal Care and Use Committee and the Institutional Review Board at the University of Virginia Health System (Protocol #3781).

**Figure 1:**
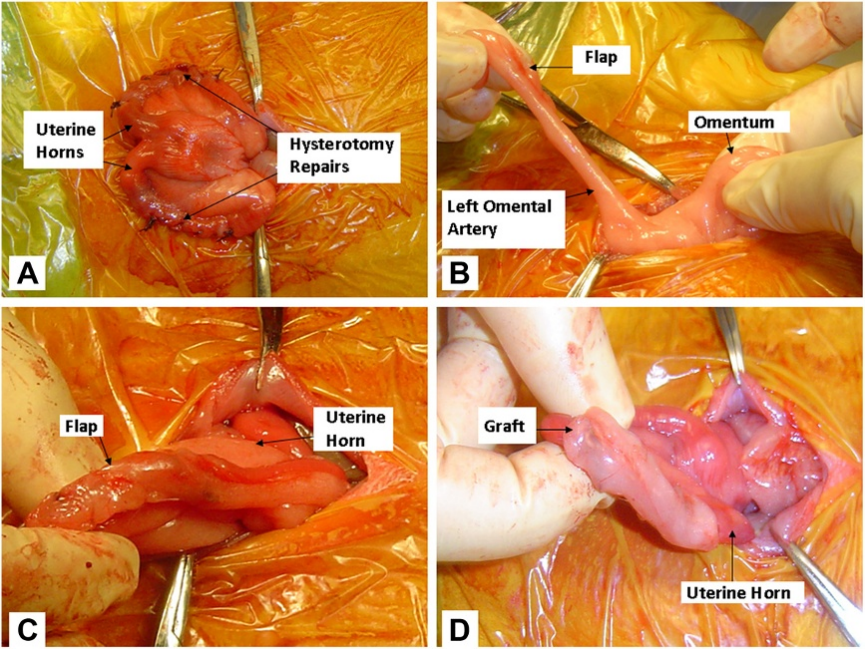
**Adhesion induction surgery. A**. Hysterotomy repair. **B**. Creation of the omental flap. **C**. Omental flap attached to uterine horn. **D**. Omental graft attached to uterine horn.

Each rabbit underwent adhesion induction surgery, the day of which was denoted as postoperative day 0. A 5 cm vertical midline infraumbilical incision was made through epidermis, subcutaneous and mammary tissue, fascia, rectus muscle and peritoneum until the peritoneal cavity was entered. The uterus was then identified and exteriorized. Electrocautery was used to make a 3 cm incision in both uterine horns, and each hysterotomy was repaired with 2–0 Vicryl suture (Ethicon Inc., Cornelia, Georgia) in a baseball stitch (Figure 1A). The right uterine horn served as the control in every rabbit. Thus, there was no omental covering over the right hysterotomy site, and it was returned to the abdomen immediately after repair of the hysterotomy. The left uterine horn in each rabbit was randomized into one of two intervention groups: coverage with an autologous free omental graft or with a pedicled omental flap. The omentum was gently pulled down from the upper abdomen and exteriorized. Then, using electrocautery, an approximately 2 cm incision was made from the right omental edge toward the midline only crossing one major artery to include adequate vasculature in the flap (Figure 1B). After the initial incision was made, the flap was rotated and sutured over the hysterotomy with seven interrupted stitches of 4–0 Monocryl (Ethicon) in a fashion that adequately covered the hysterotomy without creating tension on the omentum (Figure 1C). For the free omental graft, electrocautery was used across the inferior edge of the omentum to create an approximately 2 cm x 0.5 cm unattached piece of omentum; then this graft was placed over the hysterotomy and secured with seven interrupted stitches using 4–0 Monocryl (Figure 1D). The intervention horn was then returned to the pelvis. The hysterotomy and its repair as well as the creation and attachment to the uterus of both flaps and grafts was performed by the same surgeon (APB). We ensured that there was no tension on horns with flaps prior to closure. The fascia and rectus muscle were reapproximated in a running full-thickness stitch using 2–0 Vicryl suture. The skin was closed with 4–0 Vicryl suture with a running subcuticular stitch, and the knot was buried.

Every rabbit was randomly assigned a unique numerical identifier, which was used to distinguish each animal throughout the study. For the adhesion induction and scoring surgeries, the investigators were blinded to each rabbit’s identity and intervention group.

The rabbits were randomly divided into two groups, A and B, both of which underwent identical adhesion induction surgeries; half of each group had flaps, and half had grafts. Group A underwent look-back laparoscopy on postoperative days 2 and 8, while Group B underwent look-back procedures on postoperative days 4 and 12. This system was created to minimize the number of surgeries for each rabbit. Laparoscopies were performed using a 10 mm scope (Storz, Tuttlingen, Germany) via a single 12 mm supraumbilical (on the first laparoscopy) or left upper quadrant port (on the second laparoscopy) placed using an open technique. Laparoscopy was selected for look-back procedures to reduce the risk for additional adhesion formation from the look-back procedure itself [18]. Separate sites were used for the first and second laparoscopies in each animal to avoid damage to internal structures that might be adherent to the first laparoscopic site, a technique used in humans. A pneumoperitoneum was created using CO_2_ gas at 2 L/min flow until an intraabdominal pressure of 5-6 mmHg was obtained. Trendelenburg positioning allowed for easy viewing of each uterine horn and hysterotomy site. Adhesions were scored using a modified version of the system designed by Fiedler et al. [19] (Table 1) without including the tenacity scale in order to avoid adhesiolysis during the look-back surgeries. Scoring was done independently by two or three present surgeons (APB, AKS, DWS), and images were captured for scoring by the third surgeon when not present so that all animals had three adhesion scores averaged to determine the mean visual adhesion score.

**Table 1:** **Visual adhesion scoring system used at all look-back laparoscopies and** **at postmortem evaluation**

Characteristic	Description	Score
**Type**	**No adhesions**	**0**
	**Filmy, transparent, avascular**	**1**
	**Opaque, translucent, avascular**	**2**
	**Opaque, capillaries present**	**3**
	**Opaque, larger vessels present**	**4**
**Extent**	**No adhesions**	**0**
	**≤25% of abdomen involved**	**1**
	**≤50% of abdomen involved**	**2**
	**≤75% of abdomen involved**	**3**
	**>75% of abdomen involved**	**4**
**Inflammation**	**None**	**0**
	**Mild erythema, local surface involvement**	**1**
	**Moderate erythema, local surface involvement**	**2**
	**Severe erythema, local surface involvement**	**3**
	**Severe erythema, widespread surface involvement**	**4**

All surgeries were performed under sterile conditions after shaving and prepping the animals. Anesthesia consisted of ketamine 50 mg/kg (100 mg/mL; Fort Dodge Animal Health, Fort Dodge, Iowa) and xylazine 5 mg/kg (20 mg/mL; Butler Schein Animal Health, Dublin, Ohio) administered intramuscularly. Rabbits were masked with 100% oxygen during the procedure and monitored for hyperventilation and hypoxia. Anesthesia was maintained with isoflurane to effect. Each rabbit received antibiotic prophylaxis consisting of 22.7 mg of enrofloxacin (Bayer, Shawnee Mission, Kansas) prior to and two days after each surgery for infection prophylaxis. Rabbits were also given 0.05 mg/kg buprenorphine subcutaneously before surgery, two hours post-surgery, and twice daily for two days post-surgery to provide adequate analgesia. During look-back procedures, rabbits were given the same dosage of buprenorphine before and after the procedure along with morning and evening doses on postoperative day one. At the conclusion of each surgery, all incision sites were infiltrated with 1 mL of 0.25% bupivacaine for sustained analgesia.

On postoperative day 16, rabbits were anesthetized with 2 mL ketamine (100 mg/mL) and 1 mL xylazine (20 mg/mL) intramuscularly, and then euthanized using 1 mL of 390 mg/mL pentobarbital sodium (Virbac AH Inc., Fort Worth, Texas) by intravenous or intracardiac injection. Postmortem laparotomy and thorough inspection of the abdominal and pelvic organs for adhesion formation was performed. Again, adhesions were scored using the system designed by Fiedler et al. [19] (Table 1), this time additionally utilizing the tenacity scale (Table 2). As before, scoring was done independently by the two or three present surgeons, and images were captured for scoring by the third surgeon when not present. All scores were averaged together to create a mean visual adhesion score and mean tenacity score.

**Table 2:** **Additional tactile adhesion scoring system used at postmortem evaluation**

Characteristic	Description	Score
**Tenacity**	**No adhesions**	**0**
	**Adhesions fall apart**	**1**
	**Adhesions lysed with traction**	**2**
	**Adhesions require sharp dissection**	**3**

Statistical tests consisted of Kappa tests of agreement between adhesion scorers and Kruskal-Wallis nonparametric tests for the comparison of adhesion scores by intervention arm and by uterine horn. The statistical analysis used an alpha level of 0.05, and all analyses were conducted with SAS 9.1.3 (SAS Institute, Cary, North Carolina).

## Results

At the time of each laparoscopic look-back and at the postmortem evaluation, all flaps and grafts were noted to survive based on appearance. All flaps and grafts appeared well vascularized without discoloration or concern for tissue ischemia or necrosis. All sixteen rabbits survived every surgery, and there were very few complications. Postoperatively, one rabbit was given a single dose of 10 mg ketoprofen subcutaneously to treat additional postoperative pain, the experience of which was determined by direct observation of the rabbit. Interestingly, this rabbit had a right uterine horn that was found at the initial hysterotomy procedure to be filled with a milky fluid, but the subject remained afebrile throughout the acclimation and experimental periods. Also postoperatively, another rabbit had a ventral hernia with an associated seroma requiring a repair and look-back via laparotomy instead of laparoscopy. The data from this rabbit were discarded secondary to our concern that the adhesion formation process was compromised by the hernia and repair.

**Figure 2:**
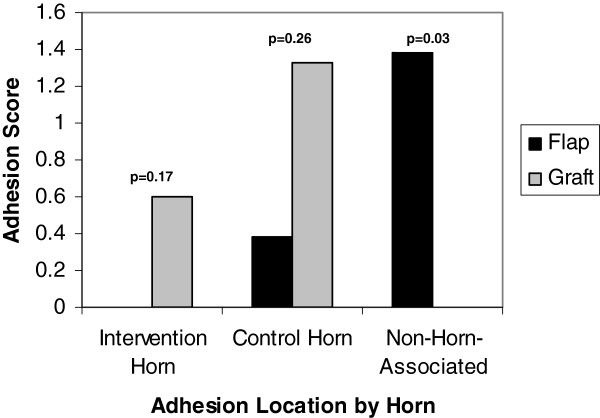
**Visual adhesion scores.** Visual adhesion scores by location and intervention at postmortem evaluation.

For the rabbits with omental flaps, the mean adhesion score for the right (control) uterine horn was 0.38 (sd 1.0, range 0–2.67) versus 0 (sd 0, range 0–0) in the left (intervention) horn, which was not statistically different (p = 0.32). For the rabbits with free omental grafts, the mean adhesion score for the control uterine horn was 1.33 (sd 2.0, range 0–5) versus 0.6 (sd 1.2, range 0–3) for the intervention uterine horn, which also was not statistically different (p = 1.00). There was no statistical difference between the adhesion scores of the flap and graft control horns (p = 0.26, Kruskal-Wallis test). There was no statistical difference between the adhesion scores of the flap and graft intervention horns (p = 0.17) (Figure 2). The absolute difference between the horns (average control uterine horn adhesion score minus average intervention uterine horn adhesion score) did not differ when comparing the flap group to the graft group on any postoperative day: 2, 4, 8, 12, or 16.

Relative to non-horn-associated adhesions in the abdomen, the rabbits with free omental grafts had no adhesions, while the rabbits with omental flaps had a mean abdominal adhesion score of 1.38 (sd 2.36, range 0–5). This difference was statistically significant (p = 0.03, Kruskal-Wallis) (Figure 2).

**Figure 3:**
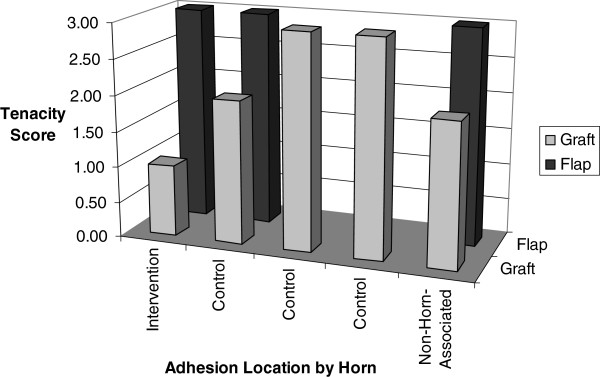
**Tenacity scores.** Tenacity scores of each adhesion by location and intervention at postmortem evaluation.

There were eight adhesions in six of the rabbits. The tenacity scores are graphed in Figure 3 by adhesion location (intervention horn, control horn, or non-horn-associated adhesion) and intervention group (flap or graft) for each adhesion. The mean tenacity score of the adhesions with omental flaps was 3.0 (sd 0, range 3–3) versus 2.2 with free omental grafts (sd 0.8, range 1–3); this was not statistically different (p = 0.12).

The agreement between the three adhesion scorers was at least “substantial” and “almost perfect” between two of the observers [20]. The kappa score ranged from 0.70 (95% CI 0.41–0.99) to 1.0 (95% CI 1.0–1.0) for the right uterine horn and was 1.0 (95% CI 1.0–1.0) for the left uterine horn.

## Discussion

The primary purpose of this pilot study was to test an animal model for the study of the omentum in pelvic adhesion prevention. We anticipated that the omental interventions might undergo necrosis and form severe adhesions, grafts more so than flaps due to absence of native blood supply. On the contrary, all omental grafts and flaps survived; therefore, this is a practical model for the study of omentum in pelvic adhesion prevention.

The secondary objective of this investigation was to compare autologous free omental grafts to pedicled omental flaps. There was no significant difference in adhesion scores between intervention and control horns or between flap and graft subjects at the hysterotomy site. There was also no significant difference in average tenacity scores of all adhesions in subjects with flaps versus those with grafts. As this was a pilot study, it was not powered to detect small differences between groups. Yet, we did see significantly fewer non-horn-associated adhesions in rabbits with free omental grafts versus those with omental flaps. If this decrease in general abdominal adhesions were to hold true in women, it would be equally as important since both fertility and operative abdominal entry can be affected by adhesions not directly attached to the uterus.

We did see a large variation in adhesion formation in the control group. This could likely be corrected if the adhesion induction surgery was performed via laparoscopy to decrease adhesions that form due to the laparotomy instead of solely from the hysterotomy.

With few rabbits forming adhesions, we are not able to say that the intervention was beneficial; but this pilot study provides a novel model with which to study the role of the omentum in pelvic adhesion prevention. The strengths of this study include its prospective nature and randomization of rabbits into intervention group. Also, the method used for hysterotomy creation mimics the procedure utilized for cesarean section hysterotomy and abdominal myomectomy, common gynecologic procedures that are known to induce adhesions. Furthermore, we had blinded adhesion scorers, used a previously validated scoring system, and our interrater agreement was substantial. The use of laparoscopy for adhesion monitoring instead of laparotomy decreased the potential for adhesion formation after the initial adhesion induction surgery; and finally, we were able to perform a thorough evaluation of the pelvis at the time of autopsy.

The primary weakness of the study is the low number of adhesions available to be scored. Future studies could incorporate more hysterotomy sites or additional thermal damage by electrocautery to induce further adhesion formation. Most adhesion formation should occur within 14 days postoperatively, so the length of the study should not be considered a weakness. Our data fit this since we did not see a significant increase in the number or severity of adhesions throughout the sixteen days.

## Conclusions

Adhesions are a known source of both short-term and long-term morbidity, and the current barrier methods for their prevention are costly and not optimally effective. In addition, some barriers are known to increase the formation of adhesions if hemostasis is not maintained. Omentum is readily available, easy to apply, noninflammatory, and inexpensive with the added ability to contain foreign matter and microbes, yet it has not been fully investigated for its potential to prevent pelvic adhesions. The survival of all flaps and grafts demonstrates that this rabbit model is an effective method for studying the omentum as a pelvic adhesion barrier. The significantly lower number of non-uterine adhesions seen with the omental grafts as compared to the flaps suggests that grafts may be a superior methodology. Future studies with a larger number of rabbits and more adhesiogenic methods should be undertaken to fully elucidate the ability of omentum to prevent pelvic adhesions.

### Availability of Supporting Data

The data sets supporting the results of this article are included within the article where they are cited and then listed in the references section.

## Author contributions

Each author has 1) made substantial contributions to conception and design, acquisition of data, and analysis and interpretation of data; 2) been involved in drafting the manuscript and revising it critically for important intellectual content; 3) given final approval of the version to be published; and 4) agrees to be accountable for all aspects of the work in ensuring that questions related to the accuracy or integrity of any part of the work are appropriately investigated and resolved. All authors read and approved the final manuscript.
